# Synthesis, characterization and photothermal analysis of nanostructured hydrides of Pd and PdCeO_2_

**DOI:** 10.1038/s41598-020-74378-1

**Published:** 2020-10-16

**Authors:** Cláudia C. R. Cruz, Nilton P. da Silva, Amanda V. Castilho, Viviane A. Favre-Nicolin, Claudio L. Cesar, Helcio R. B. Orlande, Dilson S. Dos Santos

**Affiliations:** 1 Program of Nanotechnology Engineering, COPPE, Federal University of Rio de Janeiro, Rio de Janeiro, RJ Brazil; 2 Program of Mechanical Engineering, COPPE - Oncobiology Program, Federal University of Rio de Janeiro, Rio de Janeiro, RJ Brazil; 3 Department of Mechanical Engineering, Federal University of Amazonas - UFAM, Manaus, AM Brazil; 4 Federal Institute of Education, Science and Technology of Espírito Santo - IFES, Vitoria, ES Brazil; 5 Institute of Physics, Federal University of Rio de Janeiro, Rio de Janeiro, RJ Brazil; 6 Program of Metallurgical and Materials Engineering, COPPE, Federal University of Rio de Janeiro, Rio de Janeiro, RJ Brazil

**Keywords:** Cancer therapy, Nanoscale materials, Mechanical engineering

## Abstract

Hyperthermia was shown to be an important co-adjuvant therapy to conventional cancer treatments. Nanoparticles can be used in the hyperthermia therapy to improve the localized absorption of energy imposed by external sources, in order to kill tumor cells solely by the effect of heat and with minimum thermal damage to surrounding healthy cells. Nanoparticles can also serve as carriers of drugs that specifically act on the tumor when heated, including hydrogen that can be desorbed to locally promote an antioxidant effect and reduce the viability of cancer cells. In this context, palladium hydride nanoparticles emerge as promising materials for the hyperthermia therapy. In this study, palladium nanocubes (PdNC) and PdCeO_2_ nanoparticles were synthesized. Nanofluids produced with these nanomaterials were hydrogenated and then tested to examine their photothermal effects. Nanofluids made of PdH_x_ nanoparticles presented significant temperature increases of more than 30 °C under 3 min of diode-laser irradiation. On the other hand, nanofluids with PdCeO_2_H nanoparticles presented temperature increases around 11 °C under the same experimental conditions. The behavior observed with the PdCeO_2_H nanofluids can be attributed to the effect of H^+^ in reducing Ce^+4^ to Ce^+3^.

## Introduction

Nanoparticles of noble metals and ordinary metallic alloys have been successfully used in the hyperthermia cancer therapy^1^. Nanoparticles naturally tend to concentrate in the tumor, thus locally increasing the absorption of external energy sources imposed during the hyperthermia therapy. As a result, thermal damage can be mainly imposed to the tumor cells, without significant effects on the surrounding healthy cells^2^. The use of hydrogen is very promising for the hyperthermia cancer therapy, due to its important role in physiological regulation, besides exhibiting good biomedical applicability and biosafety^3^. Hydrogen is a therapeutic antioxidant that can selectively reduce the hydroxyl radical (OH^−^), the most cytotoxic of the reactive oxygen species produced by tumor cells^4^. For the hyperthermia therapy, hydrogen must be safely and effectively stored in nanoparticles to target specific cancer cells and allow its controlled release. Nanoparticles of palladium hydride can store large amounts of hydrogen and exhibit good biocompatibility, as well as high photothermal conversion efficiencies in the near infrared range^5,6^.

Palladium and its alloys have been widely used in various engineering applications, mainly as nanocrystalline powders, especially as palladium nanocubes^7–13^. Different uses for this material have been reported, such as in catalysis^14^, sensors^15^, waste treatment^16^ and hydrogen storage^17,18^, in addition to cancer treatment^19,20^. The ability of palladium to absorb large amounts of hydrogen at room temperature and atmospheric pressure, thus forming a palladium hydride (PdH_x_), is one of its main advantages^21^. Both Pd and PdH_x_ have a fcc structure, but with different lattice parameters of 3.88 Å and 4.10 Å, respectively. Hydrogen occupies the octahedral sites of the fcc structure and diffuses through pure annealed Pd with a room temperature diffusivity of about 10^–11 ^m^2^ s^−1^^22^. Palladium hydride decomposition is slower than its formation at room temperature^23^.

When compared to their bulk versions, nanoscale metallic hydrides present faster hydrogen absorption and release reaction kinetics, due to their larger surface area and lower desorption temperature and activation energies. Thus, nanocrystalline palladium reacts fast for the formation of palladium hydride (PdH_x_). It can also release hydrogen and regenerate the metal at a high rate, as well as be recharged afterwards^24^.

Palladium nanocubes and nanoparticles of PdCeO_2_ were manufactured in this work. The hydrogen diffusivity of PdCeO_2_ is smaller than those of pure palladium in crystalline or nanocube forms. This is due to the hydrogen trap effect of CeO_2_ in the Pd matrix, which delays the hydrogen desorption. Therefore, nanoparticles made of PdCeO_2_ can potentially increase the period between production and its practical application, such as in the hyperthermia treatment. This material is also non-toxic, presents good bio-affinity and is a potent antioxidant for reactive oxygen species (ROS)^25,26^. Azambuja et al.^27^ performed the synthesis of nanostructured Pd soaked with CeO_2_ in its matrix through internal oxidation.

We note, however, that the use of palladium nanoparticles to treat diseases still has its limitations. This is due to the fact that the produced nanoparticles may contain materials that are not biocompatible, like metallic powders, salts (acetate, chloride) and other contaminants derived from the synthesis^28^.

The present work aims at synthesizing and characterizing palladium nanocubes (PdNC) and the nanostructured PdCeO_2_ alloy. It also aims at evaluating the photothermal effects of nanofluids obtained with the dispersion of these nanoparticles in distilled water. Some of the nanofluids were bubbled with hydrogen in order to form hydrides of Pd nanocubes and PdCeO_2_ nanoparticles. Nanofluids with different concentrations were continuously heated with a diode-laser (829.1 nm) during experiments with controlled powers of 154 mW, 218 mW and 439 mW.

## Results and discussion

**Figure 1:**
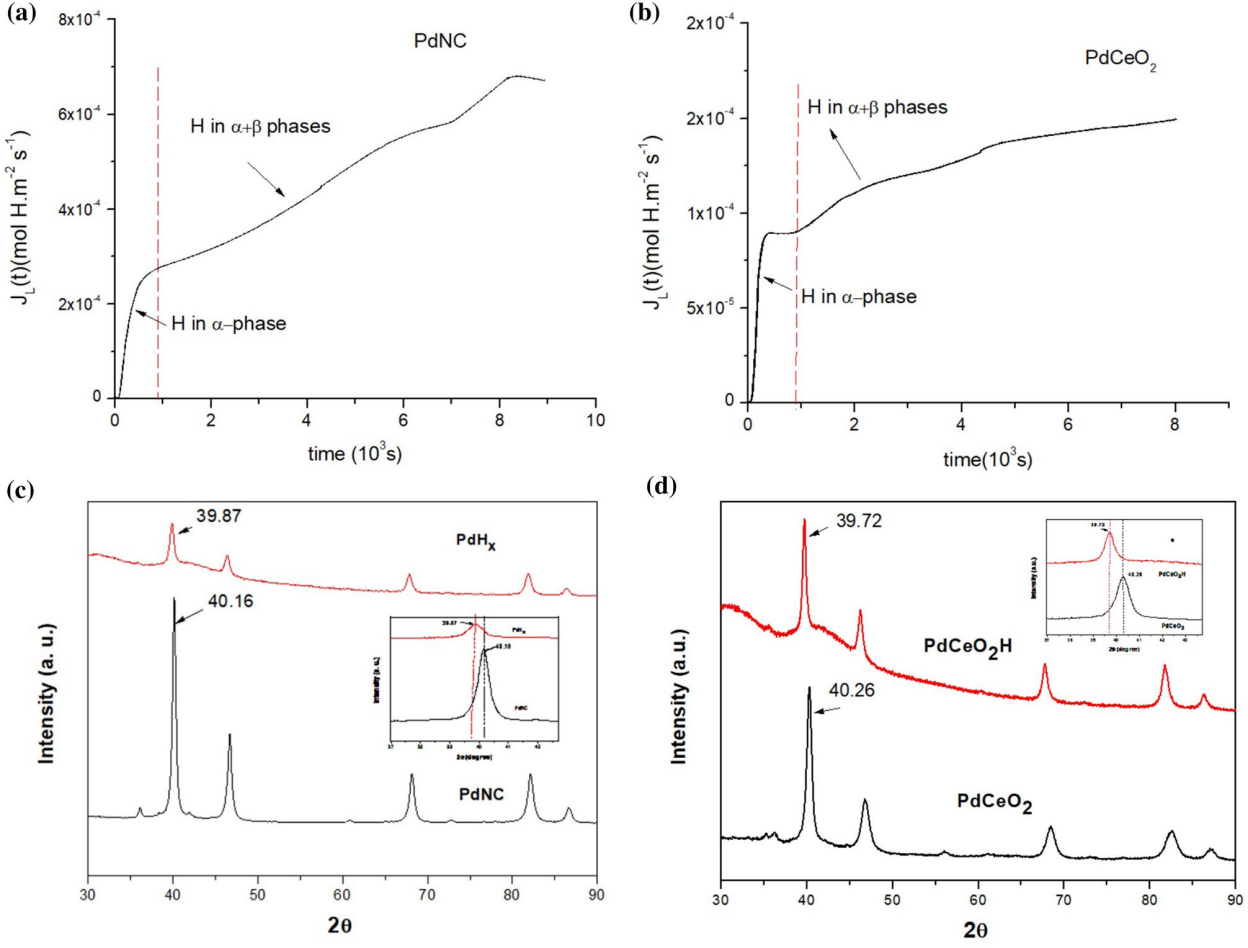
Hydrogen permeation curves obtained with current density of 5 mA/cm^2^ for pure Pd (**a**) and PdCeO_2_ internally oxidized (**b**). XRD pattern for PdNC (**c**) and PdCeO_2_ (**d**) before and after hydrogenation.

Figure 1a,b show the hydrogen permeation curves for pure Pd and PdCeO_2_, respectively, obtained at room temperature under a current density of 5 mA/cm^2^. These figures provide the hydrogen diffusivity of Pd as 3.3 × 10^–11^ m^2^ s^−1^ (Fig. 1a), which is almost six times larger than that of PdCeO_2_ (0.6 × 10^–12^ m^2^ s^−1^—see Fig. 1b). Thus, PdCeO_2_ absorbs less hydrogen during the hydrogenation period. The hydrogen diffusivity decreases with the addition of CeO_2_ in Pd, due to the accommodation of this oxide in the Pd matrix. The Ce-oxide lattice has a coincidence with a direction [111] of the Pd lattice. In this case, the interface between CeO_2_ and Pd is semi-coherent and can effectively trap hydrogen atoms. Our objective with the addition of CeO_2_ in Pd was to reduce the hydrogen diffusivity using a biocompatible material, in order to allow longer time periods between production and use of the hydrides. Besides that, its valence also plays an important role in relation to the hydrogenation, as discussed below.

Figure 1c,d show the X-ray diffraction patterns obtained for pure Pd and PdCeO_2_, respectively, before and after hydrogenation. The displacement of the peaks to the left, which represents a significant absorption of hydrogen, can be observed in Fig. 1c,d. The intensity of the diffraction peaks in the hydrogenated PdCeO_2_ alloy is lower than in the original material. Hydride precipitation, when it occurs, decreases the diffraction intensity of the primary phase. Although the hydride peaks are not clear, the shift to the left and the broadening of Pd peaks reveal the beginning of the phase transformation.

From the diffraction patterns of Fig. 1c,d it was possible to determine with Bragg’s Law the lattice parameters that are presented in Table 1. These lattice parameters reveal that the amount of hydrogen absorbed was 0.15% H/Pd for PdNC and 0.11% H/Pd for PdCeO_2_. Hydrogen at low concentrations is sufficient to form hydrides but does not necessarily form the extra peaks related to the reflection of the PdH_x_ phase. These results obtained here are in accordance with those available in the literature^3,29^.

**Table 1:** Lattice parameters for PdNC and PdCeO_2_ before and after hydrogenation.

Material	Lattice parameters (Å)
PdNC	3.89
PdH_x_	3.92
PdCeO_2_	3.88
PdCeO_2_H	3.91

**Figure 2:**
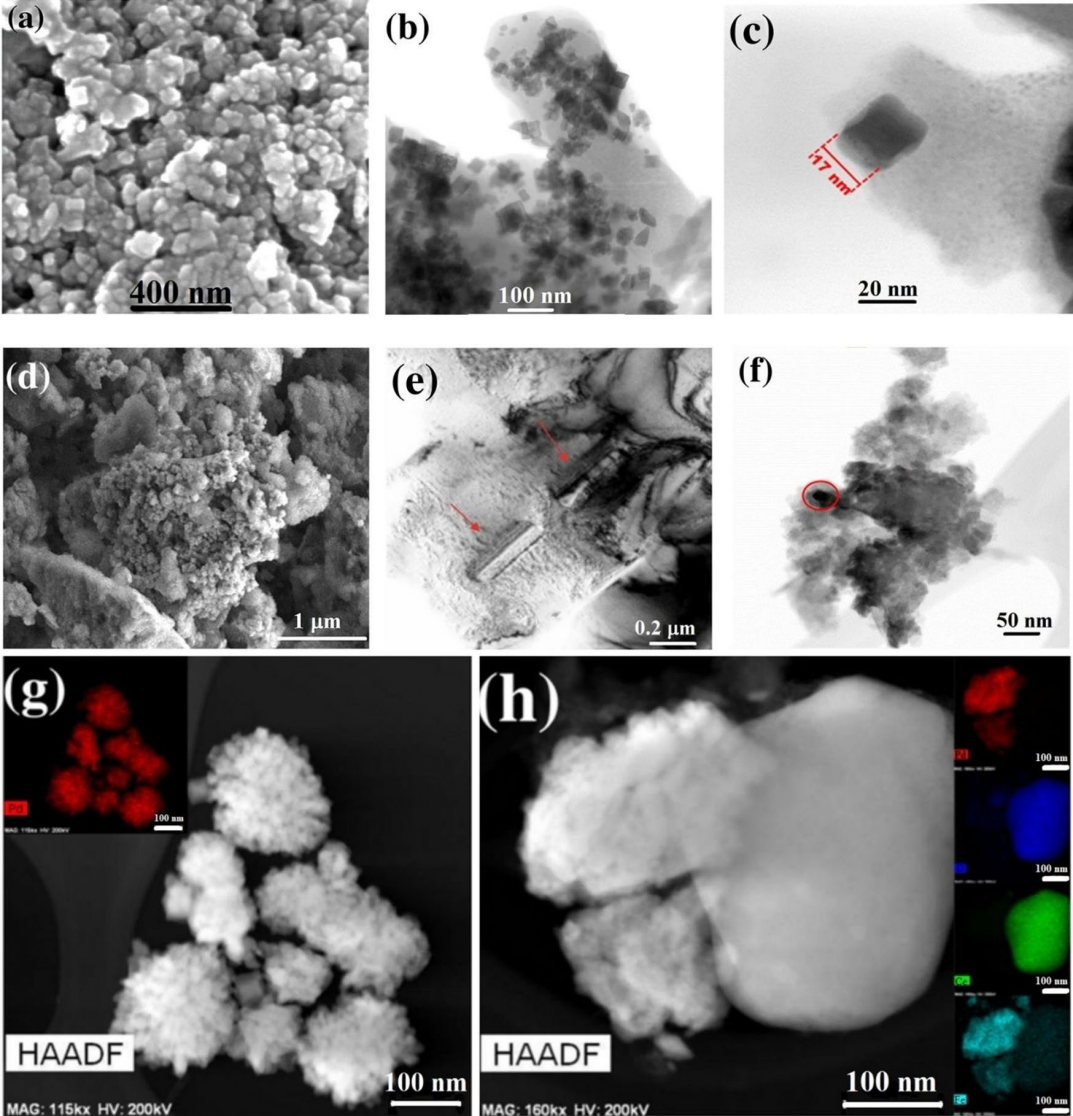
(**a**) SEM image of Pd nanocubes. (**b**) TEM image of Pd nanocubes. (**c**) TEM image of a Pd nanocube showing well-faceted interfaces. (**d**) SEM image of PdCeO_2_ alloy. (**e**) TEM image of PdCeO_2_ alloy before mechanical milling. (**f**) TEM image of PdCeO_2_ alloy after mechanical milling. (**g**) EDS mapping of Pd nanocubes. (**h**) EDS mapping of PdCeO_2_ nanoparticles. In the spotlight: Pd (red), O (blue), Ce (green) and Fe (light blue).

Figure 2a–f show the microstructural analysis for both materials obtained via SEM and TEM, respectively. The formation of Pd nanocubes with mean size 20 nm can be observed by SEM (Fig. 2a) and by TEM (Fig. 2b,c). The nano Ce oxides precipitated in the Pd matrix present nanospheres and large needles^27^. After milling, only these nanoprecipitates were observed. The CeO_2_ was always incorporated within the Pd matrix (Fig. 2e,f).

The use of CTAB in the synthesis of the nanocubes is important to stabilize the cubic morphology in the presence of bromide^30^. In addition, in a synthesis starting from Na_2_PdCl_4_ the CTAB has another important role, by reacting with the precursor and forming a complex as shown by reaction I. The addition of an excess of ascorbic acid to the colloidal complex reduces the dissociative organic salt and shifts the reaction balance slowly toward dissolution (reaction II)^31^. After mixing CTAB with Na_2_PdCl_4_, the bromide ions (Br^−^) dissociated from the surfactant can replace the chloride ions (Cl^−^) and bind to Pd^2+^, which then reacts with CTAB^+^ to form an organic salt (reaction I).

I $$ \left[ {{\text{PdCl}}_{4 - n} {\text{Br}}_{n} } \right]_{{\left( {aq} \right)}}^{ - 2} + 2{\text{CTA}}_{{\left( {aq} \right)}}^{ + } \leftrightarrow \left( {{\text{CTA}}} \right)_{2} \left[ {{\text{PdCl}}_{4 - n} {\text{Br}}_{n} } \right]_{\left( s \right)} $$

II $$ \left[ {{\text{PdCl}}_{4 - n} {\text{Br}}_{n} } \right]_{{\left( {aq} \right)}}^{ - 2} + {\text{C}}_{6} {\text{H}}_{6} {\text{O}}_{4} \left( {{\text{OH}}} \right)_{{2\,\left( {aq} \right)}} \to {\text{Pd}}\left( {{\text{NPs}}} \right) + {\text{C}}_{6} {\text{H}}_{6} {\text{O}}_{{6\,\left( {aq} \right)}} + \left( {4 - n} \right){\text{Cl}}_{{\left( {aq} \right)}}^{ - } + 2{\text{H}}_{{\left( {aq} \right)}}^{ + } + {\text{nBr}}_{{\left( {aq} \right)}}^{ - } $$

Figure 2g shows the EDS mapping of Pd nanocubes, where the presence of only palladium can be observed, free of contaminants from the organic reagents used in the reaction. Figure 2g shows the presence of clusters of Pd nanocubes. Under higher magnification, the cubic morphology becomes well defined. Figure 2h shows a CeO_2_ particle bound to Pd. In the nanostructured PdCeO_2_ alloy, the presence of iron due to the milling process was observed. Although being undesirable in the alloy, Fe would not be a concern for the hyperthermia cancer treatment application of the nanocubes, because it is a biocompatible element.

Zhao et al.^3^ presented the absorption spectrum of the palladium nanocubes and their respective hydrides up to a concentration of 0.06 g/L, in the UV–VIS–NIR, showing that the palladium nanocubes absorb more strongly in the ultra-violet (UV) range. In the presence of hydrogen, there is an increase in the absorption curve in the visible and near infrared (VIS–NIR) ranges, starting from 500 nm. Thus, the nanostructured palladium hydride has great potential for the hyperthermia therapy, since it has a large absorptivity in the NIR range and allows a controlled release of hydrogen.

**Figure 3:**
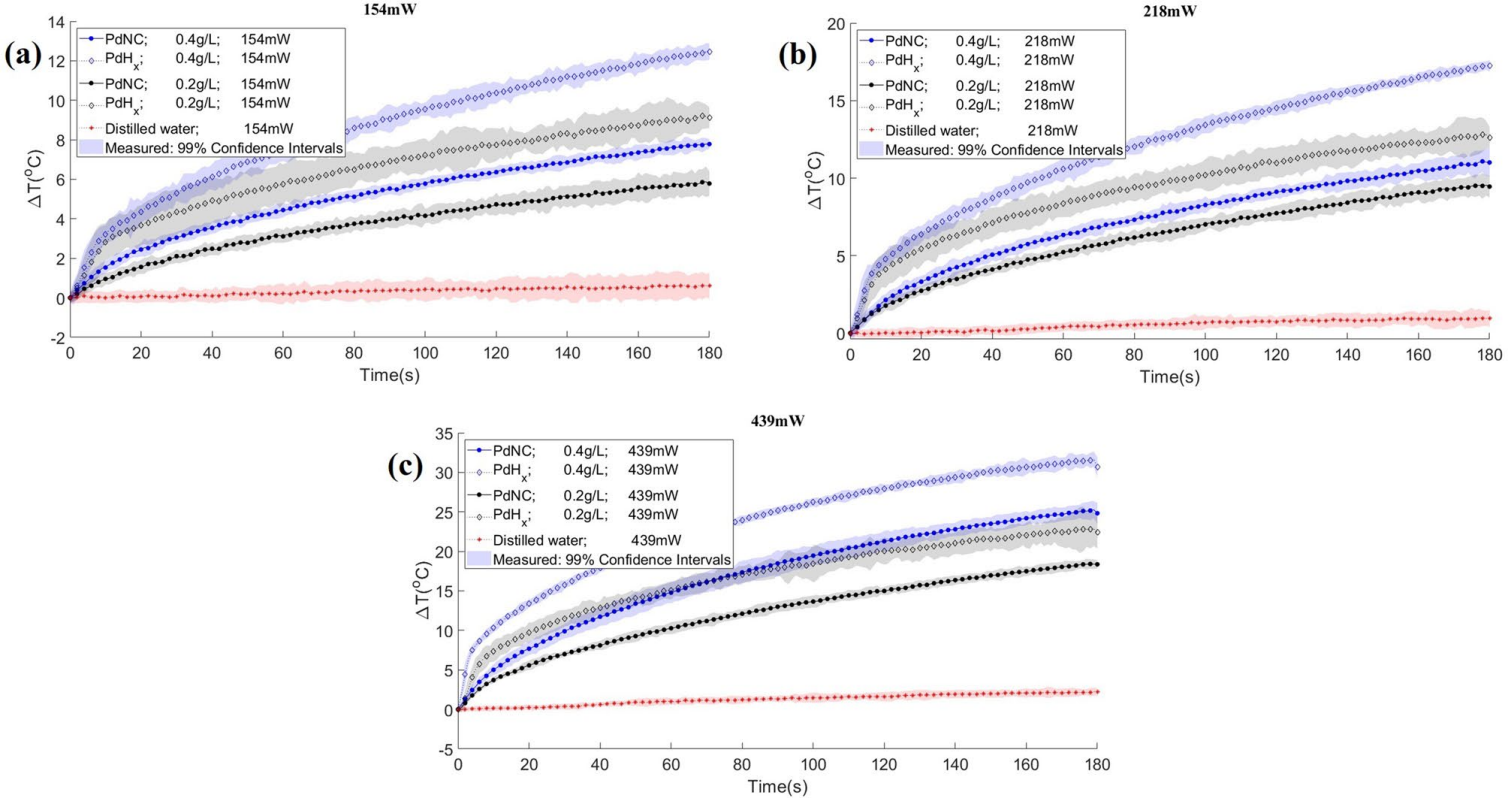
Experimental temperature variations of nanofluids made of Pd nanocubes and Pd hydride nanocubes, with concentrations of 0.2 g/L (black) and 0.4 g/L (blue). Heating performed during 3 min with three powers: (**a**) 154 mW, (**b**) 218 mW and (**c**) 439 mW. Distilled water (red) was used as white.

**Figure 4:**
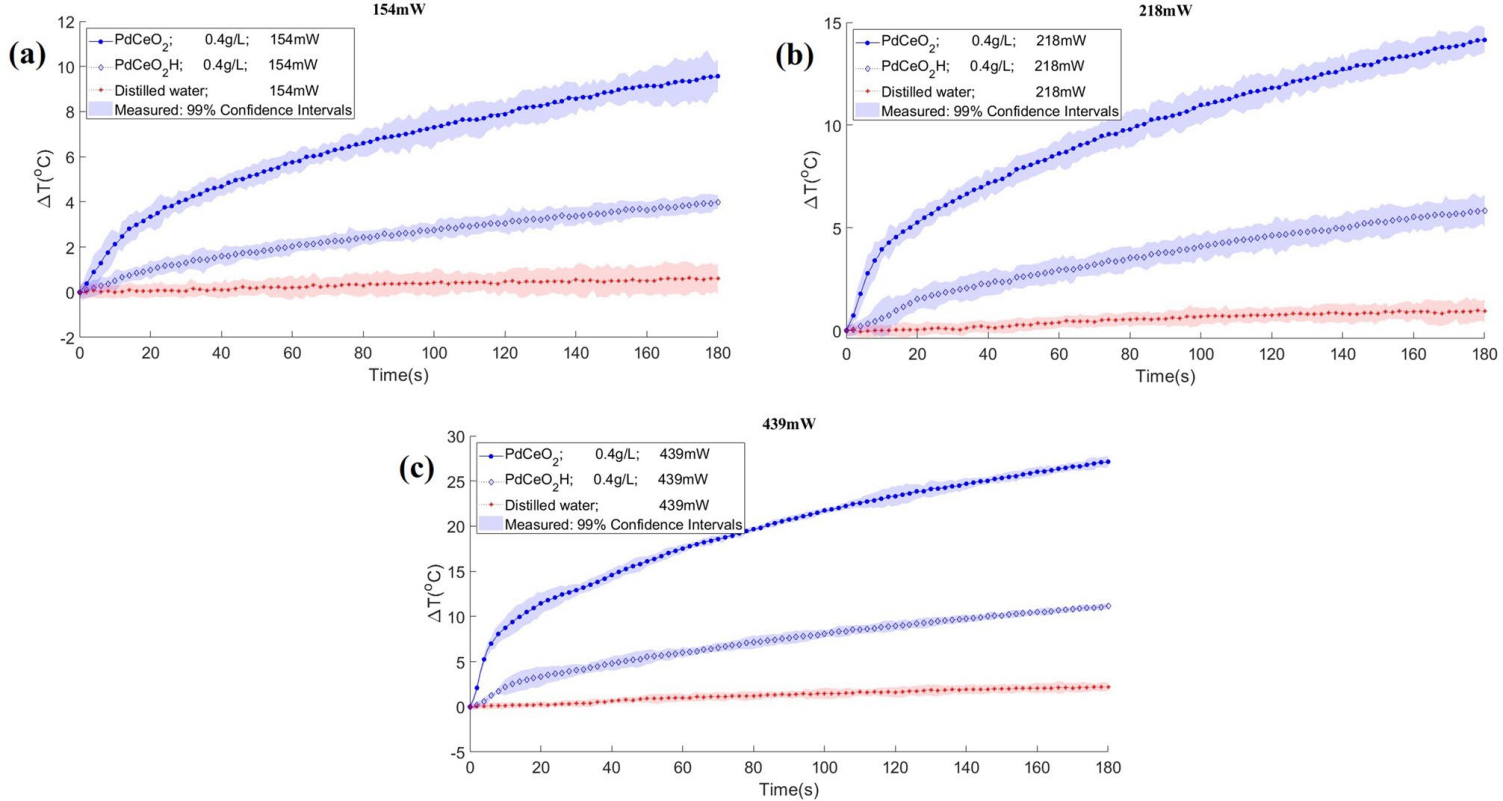
Experimental temperature variation of nano Pd cerium oxide, PdCeO_2_ (filled circle) and nano Pd hydride cerium oxide, PdCeO_2_H (filled diamond) nanofluids with a concentration of 0.4 g/L. Heating performed during 3 min with three powers: (**a**) 154 mW, (**b**) 218 mW and (**c**) 439 mW. Distilled water (red) was used as white.

In order to evaluate the photothermal effects of the nanoparticles produced in this work, distilled water nanofluids were prepared and tested under diode-laser heating, as described in the next section. Figure 3a–c present the temperature variations observed with the PdNC nanofluid and its hydrogenated version for two concentrations (0.2 and 0.4 g/L) and the powers of 154 mW, 218 mW and 439 mW, respectively. Similarly, the temperature variations observed with the PdCeO_2_ nanofluid (0.4 g/L) and its hydrogenated version are presented in Fig. 4a–c for the same diode-laser powers, respectively. The temperature variations presented in these figures were measured with an infrared camera and correspond to the pixel at the center of the fluid surface. The curves presented in Figs. 3 and 4 include the corresponding measurement uncertainties.

Figure 3a–c show that the nanofluids made with Pd hydride nanocubes exhibited much larger temperature variations than the nanofluids made with Pd nanocubes. This was expected due to the thermal effect of hydrogen during desorption. Under the laser powers of 154 mW and 218 mW, the presence of hydrogen caused larger temperature variations in the nanofluids of low concentration (e.g., Pd hydride nanocubes with concentration of 0.2 g/L under 154 mW heating presented maximum *∆T* = 9 °C) than those presented by the nanofluids of Pd nanocubes of high concentration (Pd nanocubes with concentration of 0.4 g/L under 154 mW presented maximum *∆T* = 8 °C). This demonstrates that the effect of hydrogen desorption on the temperature variation is predominant over that of the absorption cross section resulting from the nanoparticles in the fluid. At the highest power of 439 mW, the concentration of nanoparticles has a more significant effect; the larger concentrations resulted in larger temperature variations, regardless the nanoparticles (PdNC or PdH_x_) in the nanofluids. Therefore, for larger concentrations the effects of the larger absorption cross sections of the nanofluids are more important for the temperature increase than the effects of the hydrogen desorption. In addition, the curves for the nanofluids of Pd hydride nanocubes exhibited different profiles, presenting a steeper temperature variation at small times due to the presence of hydrogen, followed by profiles similar to those of the nanofluids of PdNC.

Figure 4a–c show the measured temperature variations of PdCeO_2_ and PdCeO_2_H nanofluids with a concentration of 0.4 g/L, obtained with a heating duration of 3 min and laser powers of 154 mW, 218 mW and 439 mW, respectively.

**Figure 5:**
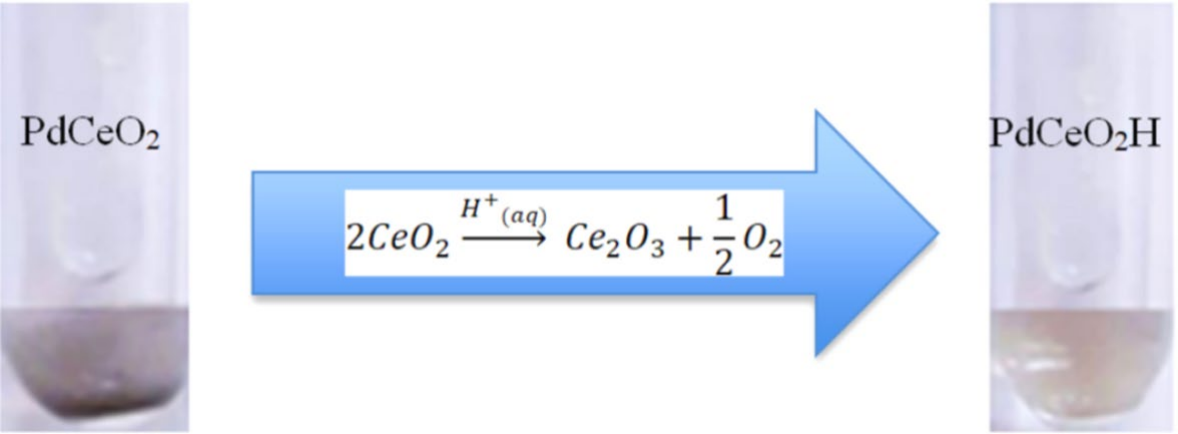
Modification of the color the PdCeO_2_ nanofluid after hydrogenation.

Table 2 summarizes the temperature variations of the nanofluids after three minutes of diode-laser heating, as shown by Figs. 3 and 4. A comparison of these temperature variations reveals that Pd nanocubes absorb less energy in the NIR than PdCeO_2_ nanoparticles. The maximum temperature variation obtained for PdNC was 25.3 °C, while for PdCeO_2_ it was 27.1 °C, for the same power of 439 mW and same concentration of 0.4 g/L. The effect of hydrogen in the nanoparticles was remarkable with PdNC, which promoted a temperature variation of 31.7 °C at the final time for the largest power and largest concentration examined, while the regular PdNC nanofluid exhibited a temperature variation of 25.3 °C for the same conditions. On the other hand, Table 2 shows that the presence of hydrogen in the nanofluids of PdCeO_2_ had the opposite effect. A reduction in the energy absorbed by the nanofluid can be noticed, which reduced the temperature variation from 27.1 °C for the PdCeO_2_ nanofluid to 11.2 °C for the PdCeO_2_ hydrogenated nanofluid. This result might have been caused by the agglomeration of the nanoparticles after hydrogenation, in addition to possible changes in valence for cerium from Ce^+4^ to Ce^+3^. In fact, there was a modification of the color of the PdCeO_2_ nanofluid when it was hydrogenated, as shown by Fig. 5.

**Table 2:** Temperature variations of nanofluids of PdNC, PdCeO_2_ and their hydrogenated forms.

Laser power (mW)	∆T (°C) after 180 s					
	PdNC 0.2 g/L	PdH_x_ 0.2 g/L	PdNC 0.4 g/L	PdH_x_ 0.4 g/L	PdCeO_2_ 0.4 g/L	PdCeO_2_H 0.4 g/L
154	5.9	9.2	7.8	12.5	9.6	4.0
218	9.5	12.8	11.1	17.3	14.1	5.8
439	18.4	22.8	25.3	31.7	27.1	11.2

As an oxide, the most stable form of cerium is Ce^+4^, where oxygen atoms occupy the tetrahedral positions and a portion of Ce^+3^ has the positive charge deficiency compensated by oxygen gaps^25^. The concentration of Ce^+3^ increases with the decrease in particle size^25^ and cerium oxide nanoparticles exhibit a significant amount of this ion. Therefore, the reacting medium will be the determining factor for the oxidation or reduction effects of cerium oxide nanoparticles. The excess of hydrogen causes Ce^+4^ to react with the hydrogen ions that were dissociated at the palladium surface, thus forming H^+^ that reduces Ce^+4^ to Ce^+3^ (reaction III)^32^. This fact modifies the absorption of the material in the NIR, resulting in a lower temperature increase than that of its non-hydrogenated form.

III $$ 2{\text{CeO}}_{2} {\mathop{\longrightarrow}\limits ^{{{\text{H}}^{ + }_{{\left( {aq} \right)}} }}} {\text{Ce}}_{2} {\text{O}}_{3} + \frac{1}{2}{\text{O}}_{2} $$

Das et al.^32^ demonstrated with XPS that Ce^+4^ and Ce^+3^ coexist in cerium oxide nanoparticles and that the presence of these two valence states on the nano-Ceria surface act as an antioxidant. Nanoparticles then eliminate the free radicals from the culture system, which gives the cerium oxide nanoparticles unique properties for biomedical use. In addition, they revealed that nano-Ceria exhibit auto-catalytic or cyclic regeneration, that is, in the presence of H_2_O_2_ it is possible to regenerate the Ce^+3^ → Ce^+4^ → Ce^+3^ system. Therefore, this material has great potential for biological applications with antioxidant activity and pseudo-infinite half-life. Hydrogenation induces the change from Ce^+4^ to Ce^+3^ by increasing the hydrogen volume^33^ and modifying material properties.

The photothermal conversion efficiencies of the nanofluids produced in this work are presented in Table 3. These values were obtained with the solution of an inverse problem by using the transient temperature variations with the laser power of 439 mW. These efficiencies follow the same trend of the temperature variations (see also Table 2). For PdNC, the efficiencies increased with the hydrogenation of the nanofluids, but the opposite behavior was observed with PdCeO_2_ nanoparticles. Also, the efficiencies increased with the concentration of PdNC. The maximum efficiency (70%) was obtained with the hydrogenated PdNC nanofluid with concentration of 0.4 g/L of PdH_x_, while the minimum efficiency (24%) was observed with the PdCeO_2_H nanofluid. The values reported in Table 3 for PdNC nanofluids are in accordance with those reported in the literature^3^. The photothermal conversion efficiencies obtained with the other laser powers were similar to those presented in Table 3, but exhibited larger standard deviations; they are not reported here for the sake of brevity.

**Table 3:** Photothermal conversion efficiencies of nanofluids of Pd, PdCeO_2_ and their hydrogenated forms with the laser power of 439 mW. Numbers inside parentheses are the standard deviations of the efficiencies.

Photothermal conversion efficiency (standard deviation), %					
PdNC 0.2 g/L	PdH_x_ 0.2 g/L	PdNC 0.4 g/L	PdH_x_ 0.4 g/L	PdCeO_2_ 0.4 g/L	PdCeO_2_H 0.4 g/L
42 (5)	55 (4)	56 (4)	70 (5)	59 (4)	24 (4)

**Figure 6:**
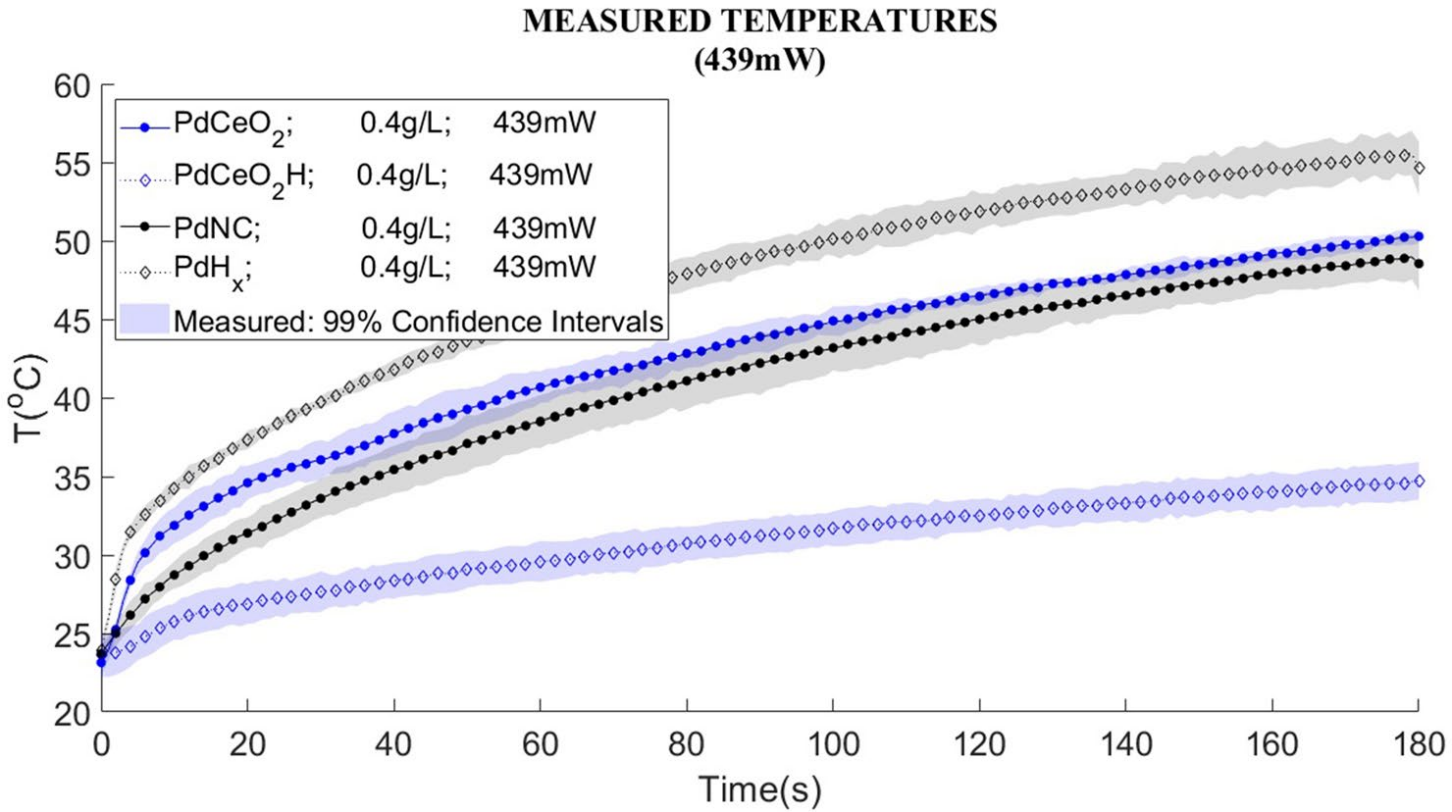
Temperature for palladium and PdCeO_2_ nanofluids.

Figure 6 summarizes the temperature variations for nanofluids of PdNC, PdCeO_2_ and their hydrogenated forms, obtained with the laser heating power of 439 mW. Although the absorption of the diode-laser energy in the hydrogenated PdCeO_2_ nanofluid was smaller than for the other nanofluids (Table 3 and Fig. 6), the resulting temperature increase might still be appropriate for the hyperthermia treatment of cancer depending on the aimed application, which may involve mild temperature increases to make the tumor cells more susceptible to chemotherapy or radiotherapy treatments.

The results obtained in the present work demonstrate the immense potential of Pd nanocubes and nanoparticles of PdCeO_2_, as well as their hydrogenated forms, in applications such as the hyperthermia therapy of cancer.

## Methods

Hydrogen permeation tests were performed at room temperature with foils about 150 µm thick, of pure palladium and PdCeO_2_. A cathodic current density of 5 mA cm^−2^, which was enough to produce hydride on the surface of the cathodic loading side, was applied to each material. In order to calculate the apparent hydrogen diffusion coefficient, electrochemical permeation tests were performed using a double-cell experiment separated by the sample^34^. An oxidation cell (detection side) was filled with NaOH 0.1 M and the electrochemical potential used was obtained from open potentiometric circuit. A reduction cell (cathodic charging side) was filled with 0.1 M H_2_SO_4_ + 2 mg/l As_2_O_3_ solution. The anodic current was detected on the cell oxidation side. Both currents were generated or detected by an AUTOLAB PGSTAT100N potentiostat.

The apparent hydrogen diffusion coefficient (*D*_*app*_) was calculated according to^18^:

1 $$ D_{app} = 0.76\frac{{L^{2} }}{{\pi^{2} t_{b} }} $$

where *D*_*app*_ is the apparent diffusion coefficient, *L* is the thickness of the sample and *t*_*b*_ is the breakthrough time, which corresponds to the time when the first hydrogen atoms permeating through the sample are detected.

For single-phase samples without hydride formation, the permeation curve is sigmoidal. When hydride reaction takes place, the hydrogen permeation curves exhibit a different evolution, which corresponds to hydride nucleation and growth during the test.

Palladium nanocubes (PdNC) were prepared through a precipitation technique using 10 mM aqueous of Na_2_PdCl_4_ (disodium tetrachloropalladate, Sigma-Aldrich 98%) and CTAB (cetyltrimethylammonium bromide, Sigma-Aldrich 98%) added to a solution 0.1 M of ascorbic acid (Sigma-Aldrich 99%). The final solution was maintained under magnetic agitation for 1 h at room temperature. After the reaction, the sample was washed several times and then air dried.

**Figure 7:**
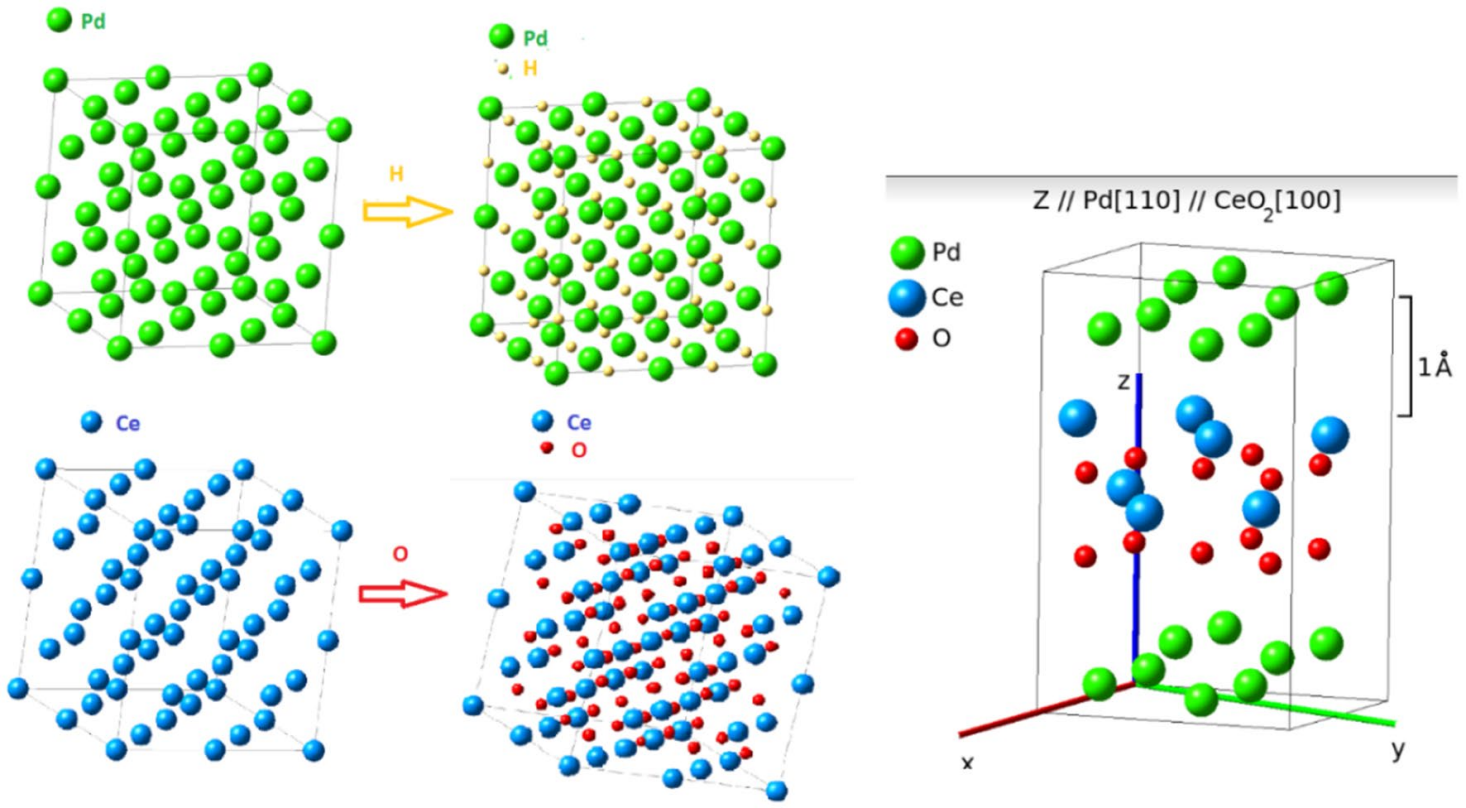
Crystalline cells of Pd and Pd-hydride, CeO_2_, PdCeO_2_ and Pd-CeO_2_ Interface obtained from MEDEA simulation software.

The alloy containing Pd and 3 wt % of Ce was melted in an arc-furnace under inert atmosphere. The alloy was then cold-rolled up to 200 µm of thickness and 15 mm × 300 mm ribbon plates were cut. These were subjected to thermal treatment at 800 °C for 24 h to promote oxygen diffusion in the Pd matrix and the formation of Ce-oxides. The oxide grows in needle or plates shapes, in accordance with the [110] direction of Pd, due to the perfect accommodation between the lattice parameters of CeO_2_ (5.42 Å) and the diagonal of the Pd cubic cell (5.50 Å) in the (110) plane, as shown in Fig. 7. After the oxidation step, the PdCeO_2_ alloy was hydrogenated and submitted to mechanical milling using a ball mill at 300 rpm for 12 h. Hydrogenation was performed to facilitate the comminution of the alloy. The hydrogen absorbed by the alloy was then desorbed during milling, due to the heating caused by friction between the balls of the mill.

An ultrasonic probe was used for dispersion of the PdNC and PdCeO_2_ nanoparticles in distilled water for 1 min. PdNC nanofluids with concentrations of 0.2 g/L and 0.4 g/L, as well as a PdCeO_2_ nanofluid with concentration of 0.4 g/L, were prepared. Half of the produced nanofluid samples were hydrogenated to form nanoparticles of metal hydride. These nanofluids were bubbled with hydrogen gas at the pressure of 2 bar and constant flow for 1 h at room temperature.

Both nanocrystalline materials were characterized by X-ray diffraction using the Cu-K_α_ radiation with wavelength of λ = 1.5406 Å. SEM analyses for morphological characterization of the nanoparticles were conducted with a SEM-FEG equipped with a FEI QUANTA detector FEG 250. TEM analyses with a FEI TECNAI G2F20 HRTEM and a TITAN G2 80-200a were also performed to determine the morphology of the materials. Identification of the chemical composition of the materials was performed via EDS analysis of fields with a TITAN G2 80-200a.

**Figure 8:**
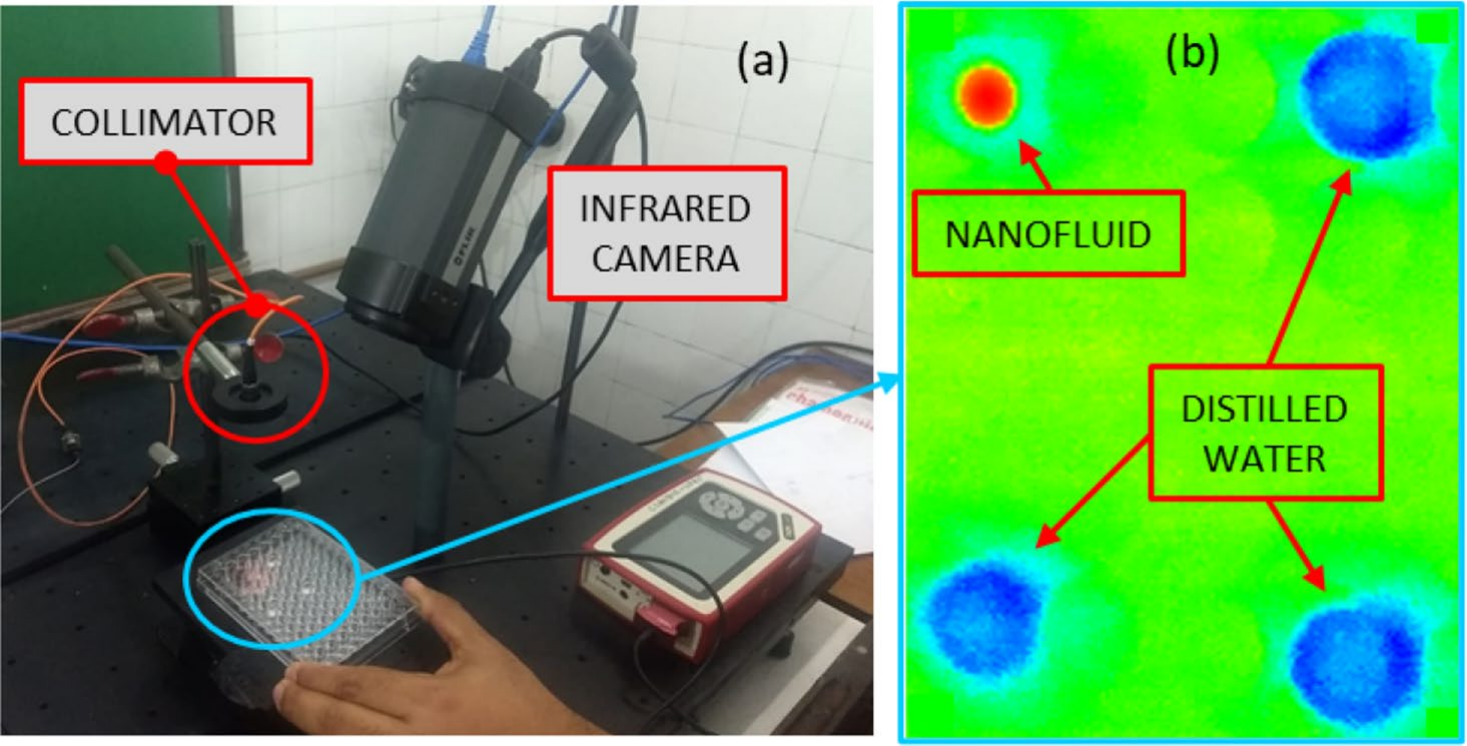
(**a**) Apparatus for photothermal analysis and (**b**) typical thermal image during the heating of the nanofluid.

Tests were performed in order to measure the temperature variation in each nanofluid and in distilled water, under the irradiation of a diode-laser. The fluids were pipetted into one well (diameter of 6.75 mm and height of 11.1 mm) of 96-well plates, with volume of 260 µL. Heat was provided during 180 s by a diode-laser (NEWPORT, model 525B at a wavelength of 829.1 nm), with collimated beam (Collimator THORLABS CFC-2X-B) and for three different powers (154 mW, 218 mW and 439 mW). The laser power was measured with an optical meter (THORLABS PM100D with Standard Photodiode Power sensor S121C) and the laser beam exhibited a Gaussian profile with 3.1 mm of diameter (THORLABS—Compact USB 2.9 CMOS Camera). The collimator was perpendicular to the 96-well plate, coaxial with the well containing the fluid and at a distance of 135 mm from the fluid surface. Temperature measurements of the surface of the fluids were taken with an infrared camera (FLIR A325), with a frequency of one measurement per second and pixel size of 0.24 mm. Figure 8a shows the apparatus used for the photothermal measurements, while Fig. 8b presents a thermal image during the laser irradiation of the well with the nanofluid. Other neighboring wells in the 96-well plate containing distilled water, which were not heated by the diode-laser but served as reference for the temperature measurements, also appear in the figure. The heating experiments were carried out in triplicates to ensure reproducibility. The curves presented for the temperature variations correspond to the mean values of the three experiments, which were conducted in a room with controlled ambient temperature (23.5 ± 0.5 °C).

The measured temperatures were also used for the calculation of the photothermal conversion efficiencies of the produced nanofluids, by following a procedure similar to that proposed by Roper et al*.*^35^. While these authors used the transient temperature variation during the cooling period and the steady-state temperature after the heating period to calculate the photothermal conversion efficiency, in this work we used an inverse analysis with the transient temperature measurements during the heating period. The model parameters were estimated with the temperature measurements in experiments with distilled water and with the produced nanofluids. The inverse parameter estimation problems were solved within the Bayesian framework of statistics by using the Markov Chain Monte Carlo (MCMC) method^36^. This method allows for the estimation of mean values and the related uncertainties of the model parameters, through stochastic simulation of the posterior probability distribution function. The values reported in this work for the photothermal conversion efficiencies are the means of the equilibrium distributions obtained with the Markov chains. The reported standard deviations of the photothermal conversion efficiencies were obtained from the standard deviations of the samples in the equilibrium Markov chains and by applying uncertainty propagation.

## Conclusions

Palladium nanocubes were synthesized via chemical precipitation in the presence of CTAB as a surfactant. The synthesis method was effective, resulting in nanocubes free of contamination and about 20 nm in size. Internal oxidation and mechanical grinding were also used to produce a nanostructured PdCeO_2_ alloy, which presented the formation of nano-Ceria in the palladium matrix. The materials were tested in experiments involving the heating of nanofluids with a diode-laser. Temperature variations of the nanofluids were substantially larger than that of distilled water, demonstrating the photothermal effect of the nanoparticles developed in this work, including those that were hydrogenated. Our results reveal a great potential for the application of these nanoparticles in the hyperthermia cancer therapy.
